# Topological Characterization of Carbon Graphite and Crystal Cubic Carbon Structures

**DOI:** 10.3390/molecules22091496

**Published:** 2017-09-07

**Authors:** Wei Gao, Muhammad Kamran Siddiqui, Muhammad Naeem, Najma Abdul Rehman

**Affiliations:** 1 School of Information Science and Technology, Yunnan Normal University, Kunming 650500, China; 2 Department of Mathematics, COMSATS Institute of Information Technology, Sahiwal 57000, Pakistan; kamransiddiqui75@gmail.com (M.K.S); naeempkn@gmail.com (M.N); najma_ar@hotmail.com (N.A.R)

**Keywords:** hyper-Zagreb index, first multiple Zagreb index, second multiple Zagreb index, Zagreb polynomials, carbon graphite, crystal structure of cubic carbon

## Abstract

Graph theory is used for modeling, designing, analysis and understanding chemical structures or chemical networks and their properties. The molecular graph is a graph consisting of atoms called vertices and the chemical bond between atoms called edges. In this article, we study the chemical graphs of carbon graphite and crystal structure of cubic carbon. Moreover, we compute and give closed formulas of degree based additive topological indices, namely hyper-Zagreb index, first multiple and second multiple Zagreb indices, and first and second Zagreb polynomials.

## 1. Introduction

Chemical graph theory has a variety of applications in the study of chemical compounds. The manipulation and examination of chemical structural information is made conceivable by using molecular descriptors. A great variety of topological indices are studied and used in the theoretical chemistry and pharmaceutical fields.

Graph theory plays the role of the mathematical part for modeling and designing of chemical structures and complex networks. The chemical graph theory applies combinatorial and geometrical graph theory to the mathematical modeling of molecular phenomena, which is helpful for the study of molecular structure. This theory contributes a prominent role in the field of chemical sciences. In graph theory, a chemical structure can be represented by vertices and edges where vertices denotes atoms and edges denotes molecular bonds. A topological index is a numeric number, which indicates some useful information about shape and analysis of molecular structure. It is the numerical invariants of a molecular graph and are useful to correlate with their bioactivity and physio-chemical properties. Over the years, researchers have found topological indices as a powerful and useful tool in the description of molecular structure. The research work in the field of chemical graph theory about the topological applications of carbon nanocones, extremal pentagonal chains, tree like polyphenylene, spiro hexagonal systems, polyphenylene dendrimer nanostars, nanostructures, and polyomino chains [1,2,3,4]. These chemical applications are motivated us to study topological descriptors and compute for some new chemical graphs.

An enormous amount of early medication tests implies that solid inner connections exist between the bio-medical and pharmacology attributes of medications and their sub-atomic structures. The hyper-Zagreb file, first numerous and second various Zagreb indices, and first and second Zagreb polynomials were characterized to be utilized as a part of the investigation of medication sub-atomic structures, which is very useful for pharmaceutical and medicinal researchers to get a handle on the organic and synthetic attributes of new medications. Such techniques are prevalently utilized in creating countries where enough cash is needed to manage the cost of the relevant chemical reagents and equipment. In our article, using methods for medicate sub-atomic structure examination and edge isolating innovation, we exhibit the hyper-Zagreb index, first multiple and second multiple Zagreb indices, and first and second Zagreb polynomials of a few broadly utilized synthetic structures that frequently show up in tranquilize molecular graphs (see details [5,6,7,8,9,10,11]).

The numerical encoding of chemical structure with topological indices is currently growing in importance in medicinal chemistry, pharmaceutical and bioinformatics. This approach allows the rapid collection, annotation, retrieval, comparison and mining of chemical structures within large databases. Topological indices can subsequently be used to seek quantitative structure–activity relationships (QSAR), which are models connecting chemical structure with biological activity. At the end of the nineteenth century, there was an explosion in the introduction and definition of new topological Indices.

As a consequence, it was recently observed that topological indices are used for unifying QSAR models with multiple targets, for DNA analysis, to study protein sequences, for 2D RNA structures, drug–protein or drug–RNA quantitative structure-binding relationship (QSBR) studies, in order to encode protein surface information and for protein interaction networks (PINs).

One of the many chemical compounds that are useful and necessary for the survival of living organisms are carbon, oxygen, hydrogen and nitrogen. These are helpful for the production of cells in the living organisms. Carbon is an essential element for human life. It is useful in the formation of proteins, carbohydrates and nucleic acids. It is vital for the growth of plants in the form of carbon dioxide. The carbon atoms can bond together in various ways, called allotropes of carbon. The well known forms are graphite and diamond. Recently, many new forms have been discovered including nanotubes, buckminster fullerene and sheets, crystal cubic structure, etc. The applications of different allotropes of carbon are discussed in detail [12,13].

Let G=(V,E) be a graph where *V* is the vertex set and *E* is the edge set of *G*. The degree d(t) of *t* is the number of edges of *G* incident with *t*. The length of a shortest path in a graph *G* is a distance d(s,t) between *s* and *t*. A graph can be represented by a polynomial, a numerical value or by matrix form. There are certain types of topological indices that are mainly eccentric based, degree based and distance based indices, etc. In this article, we deal with degree based topological indices.

In 2013, G.H. Shirdel, H.R. Pour and A.M. Sayadi [14] introduced a new degree based Zagreb index named “hyper-Zagreb index” as

(1) $$HM\left(G\right)=\sum_{uv\in E(G)}{\left[d(s)+d(t)\right]}^{2}.$$

M. Ghorbani and N. Azimi defined two new versions of Zagreb indices of a graph *G* in 2012 [15]. The first multiple Zagreb index $PM_{1}\left(G\right)$ and second multiple Zagreb index $PM_{2}\left(G\right)$ are defined as:

(2) $$PM_{1}\left(G\right)=\prod_{uv\in E(G)}\left[d\left(s\right)+d\left(t\right)\right],$$

(3) $$PM_{2}\left(G\right)=\prod_{uv\in E(G)}\left[d\left(s\right)\times d\left(t\right)\right].$$

The first Zagreb polynomial $M_{1}\left(G,x\right)$ and second Zagreb polynomial $M_{2}\left(G,x\right)$ are defined as:

(4) $$M_{1}\left(G,x\right)=\sum_{uv\in E(G)}x^{[d(s)+d(t)],}$$

(5) $$M_{2}\left(G,x\right)=\sum_{uv\in E(G)}x^{[d(s)\times d(t)].}$$

For more information for the chemical and graphical properties of HM(G), $PM_{1}\left(G\right)$, $PM_{2}\left(G\right)$ indices and $M_{1}\left(G,x\right)$, $M_{1}\left(G,x\right)$ polynomials, the reader is advised to see [15,16,17,18,19,20,21,22,23].

## 2. Structure of Carbon Graphite

**Figure 1:**
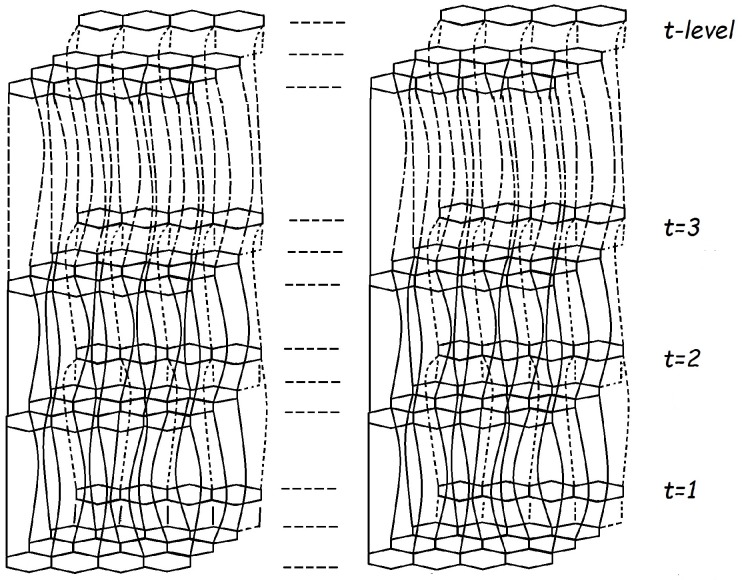
Carbon graphite CG(m,n)*t*-levels.

**Figure 2:**
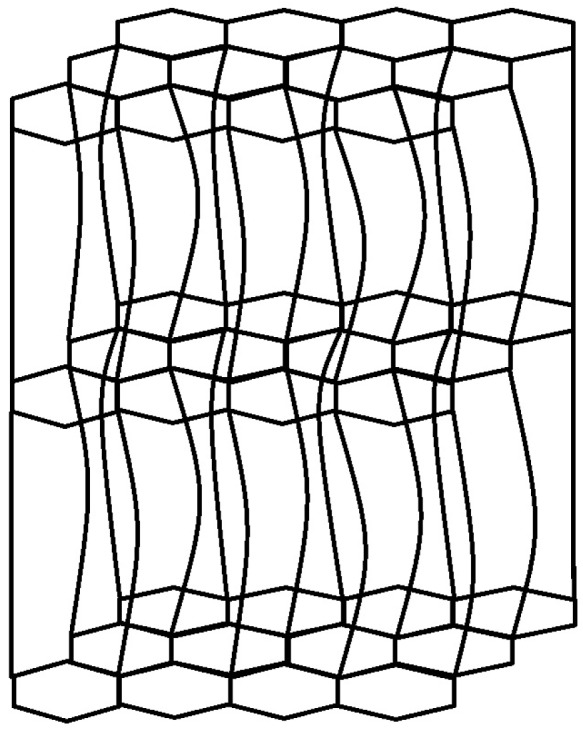
Carbon graphite graph for (3,4) and t=3.

Graphite is an allotrope of carbon. The chemical graph of carbon graphite CG(m,n) consists of layers in hexagon shapes with some weak bonding between these layers, as defined in [24]. The molecular graph of carbon graphite CG(m,n) for *t*-levels is depicted in Figure 1 and Figure 2.

The cardinality of vertices and edges in CG(m,n) are 2t(mn+m+n) and 4mnt+3mt+2nt-mn-m-t, respectively. A single layer in carbon graphite depicts the graphene layer. Carbon graphite consists of multiple layers of graphene. The *t* is the level that depicts graphene layers in carbon graphite, *m* is the number of rows and *n* is the number of columns in each layer, with *m* taken as *n* copies of hexagons in a row and *n* taken as *m* copies of hexagons in columns. [25,26,27] Ori et al., and [28], Jagadeesh discussed the topological behaviour of some graphene. In Figure 1, the levels of carbon graphite is t=3, m=3 and n=4, where m=3 is taken as n=4 copies of hexagons in a row in each level t=3, and n=4 is taken as m=3 copies of hexagons in columns in each level.

In CG(m,n), the two-degree vertices are 2(nt+m+1), the three-degree vertices are 2mn+2mt-2m+2t-4 and 4 degree vertices are 2(mn-1)(t-1). The edge set of CG(m,n) is divided into six partitions based on the degree of end vertices. Table 1 shows such an edge partition of CG(m,n) for m,n>1 and t≥2. The edge set of CG(m,n)is partitioned into four sets, say, $E_{1}$, $E_{2}$, $E_{3}$, $E_{4}$, $E_{5}$, $E_{6}$ based on the degree of end vertices of each edge. $E_{1}$ contains four edges of type uv such that d(s) = 2, d(t) = 2, $E_{2}$ contains 4(n+t-1) edges of type uv such that d(s) = 2, d(t) = 3, $E_{3}$ contains 4(nt+m-n-t) edges of type uv such that d(s) = 2, d(t) = 4, $E_{4}$ contains 4m+4t-10 edges of type uv such that d(s) = 3, d(t) = 3, $E_{5}$ contains 6mn+6mt-14m-4n-6t+12 edges of type uv such that d(s) = 3, d(t) = 4, and $E_{6}$ contains (4mn-3m-2n+1)t-7mn+5m+4n-2 edges of type uv such that d(s) = 4, d(t) = 4.

We have computed the hyper-Zagreb index HM(G), first multiple Zagreb index $PM_{1}\left(G\right)$, second multiple Zagreb index $PM_{2}\left(G\right)$ and Zagreb polynomials $M_{1}\left(G,x\right)$, $M_{2}\left(G,x\right)$ for graphite structure in the following theorems.

**Theorem** **1.** *Consider the carbon graphite CG(m,n), then its hyper Zagreb index is* HM(CG(m,n))=139+49n-97t-92nt-186m-154mn+102mt+256mnt.

**Proof.**  Let *G* be the graph of carbon graphite CG(m,n). Now by using Table 1 and Equation (1) the hyper Zagreb index are computed as:

$$\begin{array}{cc} HM(G) & =\sum_{uv\in E(G)}{\left[d\left(s\right)+d\left(t\right)\right]}^{2},\\ HM(CG(m,n)) & =\sum_{uv\in E_{1}}{\left[d\left(s\right)+d\left(t\right)\right]}^{2}+\sum_{uv\in E_{2}}{\left[d\left(s\right)+d\left(t\right)\right]}^{2}+\sum_{uv\in E_{3}}{\left[d\left(s\right)+d\left(t\right)\right]}^{2}\\ & +\sum_{uv\in E_{4}}{\left[d\left(s\right)+d\left(t\right)\right]}^{2}+\sum_{uv\in E_{5}}{\left[d\left(s\right)+d\left(t\right)\right]}^{2}+\sum_{uv\in E_{6}}{\left[d\left(s\right)+d\left(t\right)\right]}^{2},\\ HM(CG(m,n)) & =16|E_{1}\left(CG\left(m,n\right)\right)|+25|E_{2}\left(CG\left(m,n\right)\right)|\\ & +\;36|E_{3}\left(CG\left(m,n\right)\right)|+36|E_{4}\left(CG\left(m,n\right)\right)|\\ & +\;49|E_{5}\left(CG\left(m,n\right)\right)|+64|E_{6}\left(CG\left(m,n\right)\right)|,\\ HM(CG(m,n)) & =16(4)+25(4(n+t-1))+36(4(nt+m-n-t))+36(4m+4t-10)\\ & +\;49(6mn+6mt-14m-4n-6t+12)\\ & +\;64((4mn-3m-2n+1)t-7mn+5m+4n-2),\\ HM(CG(m,n)) & =139+49n-97t-92nt-186m-154mn+102mt+256mnt. \end{array}$$

☐

**Theorem** **2.** *Consider the carbon graphite CG(m,n). Then, its multiple Zagreb indices are*

$$\begin{array}{cc} PM_{1}\left(CG\left(m,n\right)\right) & =256\times 5^{4(n+t-1)}\times 6^{4(nt+m-n-t)}\times 6^{4m+4t-10}\times 7^{6mn+6mt-14m-4n-6t+12}\\ & \times \;8^{(4mn-3m-2n+1)t-7mn+5m+4n-2},\\ PM_{2}\left(CG\left(m,n\right)\right) & =256\times 6^{4(n+t-1)}\times 8^{4(nt+m-n-t)}\times 9^{4m+4t-10}\times 12^{6mn+6mt-14m-4n-6t+12}\\ & \times \;16^{(4mn-3m-2n+1)t-7mn+5m+4n-2}. \end{array}$$

**Proof.**  Let *G* be the graph of carbon graphite CG(m,n). Now, by using Table 1 and Equations (2) and (3), the multiple Zagreb indices are given by:

$$\begin{array}{cc} PM_{1}\left(G\right) & =\prod_{uv\in E(G)}\left[d\left(s\right)+d\left(t\right)\right],\\ PM_{1}\left(CG\left(m,n\right)\right) & =\prod_{uv\in E_{1}}\left[d\left(s\right)+d\left(t\right)\right]\times \prod_{uv\in E_{2}}\left[d\left(s\right)+d\left(t\right)\right]\times \prod_{uv\in E_{3}}\left[d\left(s\right)+d\left(t\right)\right]\\ & \times \;\prod_{uv\in E_{4}}\left[d\left(s\right)+d\left(t\right)\right]\times \prod_{uv\in E_{5}}\left[d\left(s\right)+d\left(t\right)\right]\times \prod_{uv\in E_{6}}\left[d\left(s\right)+d\left(t\right)\right]\\ & =4^{|E_{1}\left(CG\left(m,n\right)\right)|}\times 5^{|E_{2}\left(CG\left(m,n\right)\right)|}\times \\ & \times \;6^{|E_{3}\left(CG\left(m,n\right)\right)|}\times 6^{|E_{4}\left(CG\left(m,n\right)\right)|}\times \\ & \times \;7^{|E_{5}\left(CG\left(m,n\right)\right)|}\times 8^{|E_{6}\left(CG\left(m,n\right)\right)|,} \end{array}$$

$$\begin{array}{cc} PM_{1}\left(CG\left(m,n\right)\right) & =4^{4}\times 5^{4(n+t-1)}\times 6^{4(nt+m-n-t)}\times 6^{4m+4t-10}\times 7^{6mn+6mt-14m-4n-6t+12}\\ & \times \;8^{(4mn-3m-2n+1)t-7mn+5m+4n-2}\\ & =256\times 5^{4(n+t-1)}\times 6^{4(nt+m-n-t)}\times 6^{4m+4t-10}\times 7^{6mn+6mt-14m-4n-6t+12}\\ & \times \;8^{(4mn-3m-2n+1)t-7mn+5m+4n-2}. \end{array}$$

$$\begin{array}{cc} PM_{2}\left(G\right) & =\prod_{uv\in E(G)}\left[d\left(s\right)\times d\left(t\right)\right],\\ PM_{2}\left(CG\left(m,n\right)\right) & =\prod_{uv\in E_{1}}\left[d\left(s\right)\times d\left(t\right)\right]\times \prod_{uv\in E_{2}}\left[d\left(s\right)\times d\left(t\right)\right]\times \prod_{uv\in E_{3}}\left[d\left(s\right)\times d\left(t\right)\right]\\ & \times \;\prod_{uv\in E_{4}}\left[d\left(s\right)\times d\left(t\right)\right]\times \prod_{uv\in E_{5}}\left[d\left(s\right)\times d\left(t\right)\right]\times \prod_{uv\in E_{5}}\left[d\left(s\right)\times d\left(t\right)\right]\\ & =4^{|E_{1}\left(CG\left(m,n\right)\right)}\times 6^{|E_{2}\left(CG\left(m,n\right)\right)|}\times \\ & \times \;8^{|E_{3}\left(CG\left(m,n\right)\right)|}\times 9^{|E_{4}\left(CG\left(m,n\right)\right)|}\times \\ & \times \;12^{|E_{5}\left(CG\left(m,n\right)\right)|}\times 16^{|E_{6}\left(CG\left(m,n\right)\right)|}\\ & =4^{4}\times 6^{4(n+t-1)}\times 8^{4(nt+m-n-t)}\times 9^{4m+4t-10}\times 12^{6mn+6mt-14m-4n-6t+12}\\ & \times \;16^{(4mn-3m-2n+1)t-7mn+5m+4n-2}\\ & =256\times 6^{4(n+t-1)}\times 8^{4(nt+m-n-t)}\times 9^{4m+4t-10}\times 12^{6mn+6mt-14m-4n-6t+12}\\ & \times \;16^{(4mn-3m-2n+1)t-7mn+5m+4n-2}. \end{array}$$

☐

**Theorem** **3.** *Consider the carbon graphite CG(m,n), then its Zagreb polynomials are*

$$\begin{array}{cc} M_{1}\left(CG\left(m,n\right),x\right) & =4x^{4}+\left(4\left(n+t-1\right)\right)x^{5}+\left(4\left(nt+m-n-t\right)\right)x^{6}+\left(4m+4t-10\right)x^{6}\\ & +\;\left(6mn+6mt-14m-4n-6t+12\right)x^{7}\\ & +\;\left[\left(4mn-3m-2n+1\right)t-7mn+5m+4n-2\right]x^{8},\\ M_{2}\left(CG\left(m,n\right),x\right) & =4x^{4}+\left(4\left(n+t-1\right)\right)x^{6}+\left(4\left(nt+m-n-t\right)\right)x^{8}+\left(4m+4t-10\right)x^{9}\\ & +\;\left(6mn+6mt-14m-4n-6t+12\right)x^{12}\\ & +\;\left(\left(4mn-3m-2n+1\right)t-7mn+5m+4n-2\right)x^{16}. \end{array}$$

**Proof.**  Let *G* be the graph of carbon graphite CG(m,n). Now, by using Table 1 and Equations (4) and (5), the Zagreb polynomials are:

$$\begin{array}{cc} M_{1}\left(G,x\right) & =\sum_{uv\in E(G)}x^{[d(s)+d(t)],}\\ M_{1}\left(CG\left(m,n\right),x\right) & =\sum_{uv\in E_{1}}x^{\left[d(s)+d(t)\right]}+\sum_{uv\in E_{2}}x^{\left[d(s)+d(t)\right]}+\sum_{uv\in E_{3}}x^{\left[d(s)+d(t)\right]}+\sum_{uv\in E_{4}}x^{\left[d(s)+d(t)\right]}\\ & +\;\sum_{uv\in E_{5}}x^{\left[d(s)+d(t)\right]}+\sum_{uv\in E_{6}}x^{[d(s)+d(t)],}\\ M_{1}\left(CG\left(m,n\right),x\right) & =\;|E_{1}\left(CG\left(m,n\right)\right)|x^{4}+|E_{2}\left(CG\left(m,n\right)\right)|x^{5}+\\ & +\;|E_{3}\left(CG\left(m,n\right)\right)|x^{6}+|E_{4}\left(CG\left(m,n\right)\right)|x^{6}\\ & +\;|E_{5}\left(CG\left(m,n\right)\right)|x^{7}+|E_{6}\left(CG\left(m,n\right)\right)|x^{8},\\ M_{1}\left(CG\left(m,n\right),x\right) & =\left(4\right)x^{4}+\left(4\left(n+t-1\right)\right)x^{5}+\left(4\left(nt+m-n-t\right)\right)x^{6}+\left(4m+4t-10\right)x^{6}\\ & +\;\left(6mn+6mt-14m-4n-6t+12\right)x^{7}\\ & +\;\left(\left(4mn-3m-2n+1\right)t-7mn+5m+4n-2\right)x^{8}, \end{array}$$

$$\begin{array}{cc} M_{2}\left(G,x\right) & =\sum_{uv\in E(G)}x^{[d(s)\times d(t)],}\\ M_{2}\left(CG\left(m,n\right),x\right) & =\sum_{uv\in E_{1}}x^{\left[d(s)\times d(t)\right]}+\sum_{uv\in E_{2}}x^{\left[d(s)\times d(t)\right]}+\sum_{uv\in E_{3}}x^{\left[d(s)\times d(t)\right]}\\ & +\;\sum_{uv\in E_{4}}x^{\left[d(s)\times d(t)\right]}+\sum_{uv\in E_{5}}x^{\left[d(s)\times d(t)\right]}+\sum_{uv\in E_{6}}x^{[d(s)\times d(t)],}\\ M_{2}\left(CG\left(m,n\right),x\right) & =\;|E_{1}\left(CG\left(m,n\right)\right)|x^{4}+|E_{2}\left(CG\left(m,n\right)\right)|x^{6}\\ & +\;|E_{3}\left(CG\left(m,n\right)\right)|x^{8}+|E_{4}\left(CG\left(m,n\right)\right)|x^{9}\\ & +\;|E_{5}\left(CG\left(m,n\right)\right)|x^{12}+|E_{6}\left(CG\left(m,n\right)\right)|x^{16}\\ & =\left(4\right)x^{4}+\left(4\left(n+t-1\right)\right)x^{6}+\left(4\left(nt+m-n-t\right)\right)x^{8}+\left(4m+4t-10\right)x^{9}\\ & +\;\left(6mn+6mt-14m-4n-6t+12\right)x^{12}\\ & +\;\left(\left(4mn-3m-2n+1\right)t-7mn+5m+4n-2\right)x^{16}. \end{array}$$

☐

## 3. Crystal Structure Cubic Carbon

**Figure 3:**
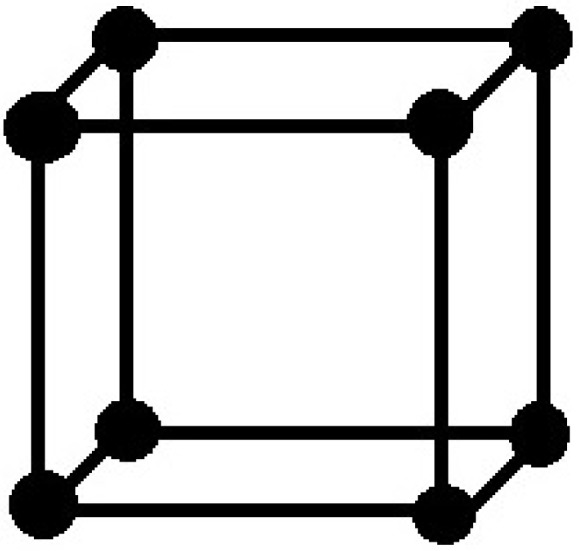
Crystal structure cubic carbon CCC(1).

**Figure 4:**
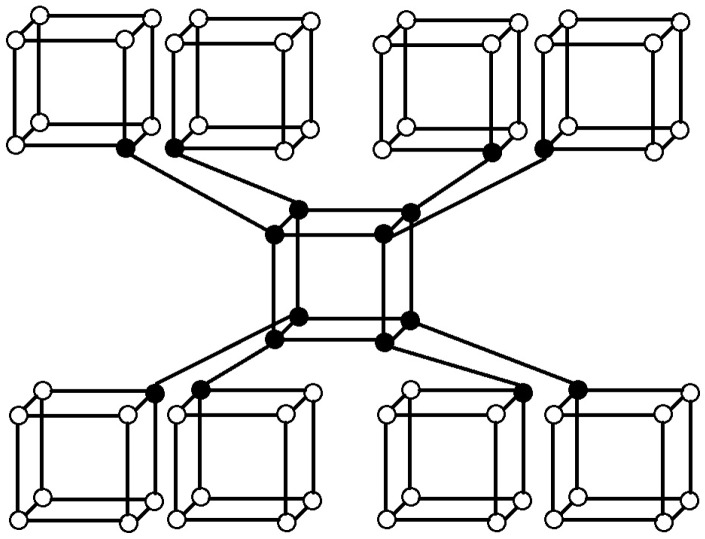
Crystal structure cubic carbon CCC(2).

Carbon is capable of forming many allotropes due to its valency. Well-known forms of carbon include diamond, graphite and one of its hypothetical allotrope is called crystal cubic carbon, which is also known as pcb. For our convenience, we take the structure of crystal cubic carbon (see [24]). The molecular graph of crystal cubic carbon CCC(n) for first layer (iteration) is depicted in Figure 3. For second layer(iteration), a new cube is attached to every cube end vertex of the first level. The second level of CCC(n) is depicted in Figure 4. Similarly, we continue this procedure to get the next level and so on. The cardinality of vertices and edges in CCC(n) are given below, respectively:

$$\left|V\left(CCC\left(n\right)\right)\right|=2\left(24\sum_{r=3}^{n}{\left(2^{3}-1\right)}^{r-3}+31{\left(2^{3}-1\right)}^{n-2}+2\sum_{r=0}^{n-2}{\left(2^{3}-1\right)}^{r}+3\right),$$

$$\left|E\left(CCC\left(n\right)\right)\right|=4\left(24\sum_{r=3}^{n}{\left(2^{3}-1\right)}^{r-3}+24{\left(2^{3}-1\right)}^{n-2}+2\sum_{r=0}^{n-2}{\left(2^{3}-1\right)}^{r}+3\right).$$

In CCC(n), the three-degree vertices are $deg\left(3\right)=8{\left(2^{3}-1\right)}^{n-1}$ and the four-degree vertices are $deg\left(4\right)=2\left(24\sum_{r=3}^{n}{\left(2^{3}-1\right)}^{r-3}+3\left({\left(2^{3}-1\right)}^{n-2}+1\right)+2\sum_{r=0}^{n-2}{\left(2^{3}-1\right)}^{r}+3\right)$. The *r* is the level of crystal cubic carbon, where 3≤r≤n. The edge set of CCC(n) is divided into three partitions based on the degree of end vertices. Table 2 shows such an edge partition of CCC(n) with n≥2. The edge set of CCC(n) is partitioned into four sets, say, $E_{1}$, $E_{2}$, $E_{3}$ based on the degree of end vertices of each edge. The set $E_{1}$ contains $72\left(2^{3}-1\right)n^{-2}$ edges of type uv such that d(s)=3, d(t)=3, $E_{2}$ contains $24\left(2^{3}-1\right)n^{-2}$ edges of type uv such that d(s)=3, d(t)=4, $E_{3}$ contains $12\left(1+\sum_{r=3}^{n}2^{3}{\left(2^{3}-1\right)}^{r-3}\right)+8\sum_{r=0}^{n-2}{\left(2^{3}-1\right)}^{r}$ edges of type uv such that d(s)=4, d(t)=4.

We have computed hyper-Zagreb index HM(G), first multiple Zagreb index $PM_{1}\left(G\right)$, second multiple Zagreb index $PM_{2}\left(G\right)$ and Zagreb polynomials $M_{1}\left(G,x\right)$, $M_{2}\left(G,x\right)$ for crystal structure cubic carbon CCC(n) in the following theorems.

**Theorem** **4.** *Considering the crystal structure cubic carbon CCC(n), n≥2, then its hyper Zagreb index is given by:*

$$\begin{array}{ccc} HM(CCC(n)) & = & 768+3768\left(\left(2^{3}-1\right)n^{-2}\right)+768\left(\sum_{r=3}^{n}2^{3}{\left(2^{3}-1\right)}^{r-3}\right)+512\sum_{r=0}^{n-2}{\left(2^{3}-1\right)}^{r}. \end{array}$$

**Proof.**  Let *G* be the graph of crystal structure cubic carbon CCC(n). Now, using Table 2 and Equation (1) the hyper Zagreb index of CCC(n) is given by:

$$\begin{array}{cc} HM(G) & =\sum_{uv\in E(G)}{\left[d\left(s\right)+d\left(t\right)\right]}^{2},\\ HM(CCC(n)) & =\sum_{uv\in E_{1}}{\left[d\left(s\right)+d\left(t\right)\right]}^{2}+\sum_{uv\in E_{2}}{\left[d\left(s\right)+d\left(t\right)\right]}^{2}+\sum_{uv\in E_{3}}{\left[d\left(s\right)+d\left(t\right)\right]}^{2}\\ & =36|E_{1}\left(CCC\left(n\right)\right)|+49|E_{2}\left(CCC\left(n\right)\right)|+64|E_{3}\left(CCC\left(n\right)\right)|\\ & =36\left(72\left(2^{3}-1\right)n^{-2}\right)+49\left(24\left(2^{3}-1\right)n^{-2}\right)+\\ & +\;64\left(12\left(1+\sum_{r=3}^{n}2^{3}{\left(2^{3}-1\right)}^{r-3}\right)+8\sum_{r=0}^{n-2}{\left(2^{3}-1\right)}^{r}\right)\\ & =768+3768\left(\left(2^{3}-1\right)n^{-2}\right)+768\left(\sum_{r=3}^{n}2^{3}{\left(2^{3}-1\right)}^{r-3}\right)+512\sum_{r=0}^{n-2}{\left(2^{3}-1\right)}^{r}. \end{array}$$

☐

**Theorem** **5.** *Considering the crystal structure cubic carbon CCC(n), n≥2, then its multiple Zagreb indices and Zagreb polynomials are*

$$\begin{array}{ccc} PM_{1}\left(CCC\left(n\right)\right) & = & 6^{72\left(2^{3}-1\right)n^{-2}}\times 7^{24\left(2^{3}-1\right)n^{-2}}\times 8^{12\left(1+\sum_{r=3}^{n}2^{3}{\left(2^{3}-1\right)}^{r-3}\right)+8\sum_{r=0}^{n-2}{\left(2^{3}-1\right)}^{r}},\\ PM_{2}\left(CCC\left(n\right)\right) & = & 9^{72\left(2^{3}-1\right)n^{-2}}\times 12^{24\left(2^{3}-1\right)n^{-2}}\times 16^{12\left(1+\sum_{r=3}^{n}2^{3}{\left(2^{3}-1\right)}^{r-3}\right)+8\sum_{r=0}^{n-2}{\left(2^{3}-1\right)}^{r}}. \end{array}$$

**Proof.**  Let *G* be the graph of crystal structure cubic carbon CCC(n). Now, using Table 2 and Equations (2) and (3), the multiple Zagreb indices of CCC(n) are computed below:

$$\begin{array}{cc} PM_{1}\left(G\right) & =\prod_{uv\in E(G)}\left[d\left(s\right)+d\left(t\right)\right],\\ PM_{1}\left(CCC\left(n\right)\right) & =\prod_{uv\in E_{1}}\left[d\left(s\right)+d\left(t\right)\right]\times \prod_{uv\in E_{2}}\left[d\left(s\right)+d\left(t\right)\right]\times \prod_{uv\in E_{3}}\left[d\left(s\right)+d\left(t\right)\right]\times \\ & =6^{|E_{1}\left(CCC\left(n\right)\right)|}\times 7^{|E_{2}\left(CCC\left(n\right)\right)|}\times 8^{|E_{3}\left(CCC\left(n\right)\right)|}\\ & =6^{72\left(2^{3}-1\right)n^{-2}}\times 7^{24\left(2^{3}-1\right)n^{-2}}\times \\ & \times \;8^{12\left(1+\sum_{r=3}^{n}2^{3}{\left(2^{3}-1\right)}^{r-3}\right)+8\sum_{r=0}^{n-2}{\left(2^{3}-1\right)}^{r}}. \end{array}$$

$$\begin{array}{cc} PM_{2}\left(G\right) & =\prod_{uv\in E(G)}\left[d\left(s\right)\times d\left(t\right)\right],\\ PM_{2}\left(CCC\left(n\right)\right) & =\prod_{uv\in E_{1}}\left[d\left(s\right)\times d\left(t\right)\right]\times \prod_{uv\in E_{2}}\left[d\left(s\right)\times d\left(t\right)\right]\times \prod_{uv\in E_{3}}\left[d\left(s\right)\times d\left(t\right)\right]\times \\ & =9^{|E_{1}\left(CCC\left(n\right)\right)|}\times 12^{|E_{2}\left(CCC\left(n\right)\right)|}\times 16^{|E_{3}\left(CCC\left(n\right)\right)|}\\ & =9^{72\left(2^{3}-1\right)n^{-2}}\times 12^{24\left(2^{3}-1\right)n^{-2}}\times 16^{12\left(1+\sum_{r=3}^{n}2^{3}{\left(2^{3}-1\right)}^{r-3}\right)+8\sum_{r=0}^{n-2}{\left(2^{3}-1\right)}^{r}}. \end{array}$$

☐

**Theorem** **6.** *Considering the crystal structure cubic carbon CCC(n), n≥2, then its multiple zagreb indices and zagreb polynomials are*

$$\begin{array}{cc} PM_{1}\left(CCC\left(n\right),x\right) & =\left(72\left(2^{3}-1\right)n^{-2}\right)x^{6}+\left(24\left(2^{3}-1\right)n^{-2}\right)x^{7}\\ & +\;\left(12\left(1+\sum_{r=3}^{n}2^{3}{\left(2^{3}-1\right)}^{r-3}\right)+8\sum_{r=0}^{n-2}{\left(2^{3}-1\right)}^{r}\right)x^{8},\\ PM_{2}\left(CCC\left(n\right),x\right) & =\left(72\left(2^{3}-1\right)n^{-2}\right)x^{9}+\left(24\left(2^{3}-1\right)n^{-2}\right)x^{12}+\\ & +\;\left(12\left(1+\sum_{r=3}^{n}2^{3}{\left(2^{3}-1\right)}^{r-3}\right)+8\sum_{r=0}^{n-2}{\left(2^{3}-1\right)}^{r}\right)x^{16}. \end{array}$$

**Proof.**  Let *G* be the graph of crystal structure cubic carbon CCC(n). Now, using Table 2 and Equations (4) and (5), the Zagreb polynomials of CCC(n) is given by:

$$\begin{array}{cc} M_{1}\left(G,x\right) & =\sum_{uv\in E(G)}x^{[d(s)+d(t)],}\\ M_{1}\left(CCC\left(n\right),x\right) & =\sum_{uv\in E_{1}}x^{\left[d(s)+d(t)\right]}+\sum_{uv\in E_{2}}x^{\left[d(s)+d(t)\right]}+\sum_{uv\in E_{3}}x^{[d(s)+d(t)],}\\ M_{1}\left(CCC\left(n\right),x\right) & =\;|E_{1}\left(CCC\left(n\right)\right)|x^{6}+|E_{2}\left(CCC\left(n\right)\right)|x^{7}+|E_{3}\left(CCC\left(n\right)\right)|x^{8},\\ M_{1}\left(CCC\left(n\right),x\right) & =\left(72\left(2^{3}-1\right)n^{-2}\right)x^{6}+\left(24\left(2^{3}-1\right)n^{-2}\right)x^{7}+\\ & +\;\left(12\left(1+\sum_{r=3}^{n}2^{3}{\left(2^{3}-1\right)}^{r-3}\right)+8\sum_{r=0}^{n-2}{\left(2^{3}-1\right)}^{r}\right)x^{8}, \end{array}$$

$$\begin{array}{cc} M_{2}\left(G,x\right) & =\sum_{uv\in E(G)}x^{[d(s)\times d(t)],}\\ M_{2}\left(CCC\left(n\right),x\right) & =\sum_{uv\in E_{1}}x^{\left[d(s)\times d(t)\right]}+\sum_{uv\in E_{2}}x^{\left[d(s)\times d(t)\right]}+\sum_{uv\in E_{3}}x^{\left[d(s)\times d(t)\right]}+\\ M_{2}\left(CCC\left(n\right),x\right) & =|E_{1}\left(CCC\left(n\right)\right)|x^{9}+|E_{2}\left(CCC\left(n\right)\right)|x^{12}+|E_{3}\left(CCC\left(n\right)\right)|x^{16}\\ & =\left(72\left(2^{3}-1\right)n^{-2}\right)x^{9}+\left(24\left(2^{3}-1\right)n^{-2}\right)x^{12}+\\ & +\;\left(12\left(1+\sum_{r=3}^{n}2^{3}{\left(2^{3}-1\right)}^{r-3}\right)+8\sum_{r=0}^{n-2}{\left(2^{3}-1\right)}^{r}\right)x^{16}. \end{array}$$

☐

## 4. Conclusions

In this paper, we deal with CG(m,n), CCC(n) and studied their topological indices. We determined the hyper-Zagreb index HM(G), first multiple Zagreb index $PM_{1}\left(G\right)$, second multiple Zagreb index $PM_{2}\left(G\right)$ and Zagreb polynomials $M_{1}\left(G,x\right)$ , $M_{2}\left(G,x\right)$.

**Figure 5:**
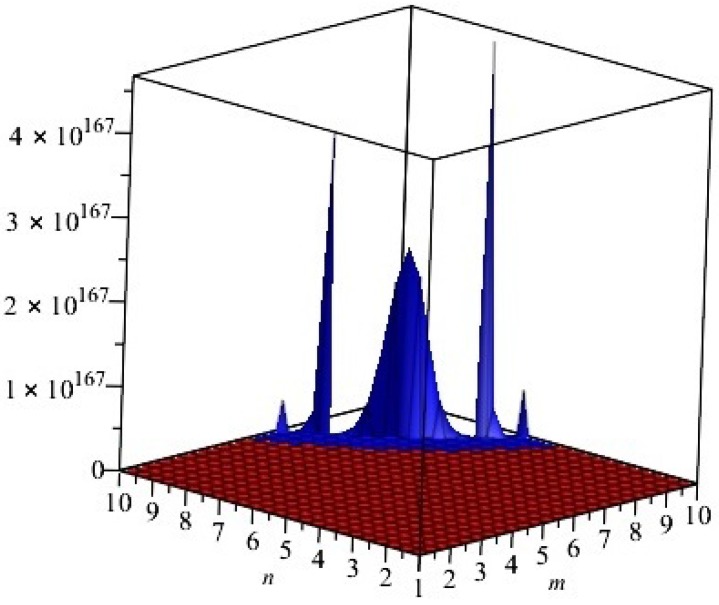
First and second multiple Zagreb indices $PM_{1}\left(G\right)$ and $PM_{2}\left(G\right)$ of *G* equivalent to CG(m,n), for t=1. Blue and red colors represent $PM_{1}\left(G\right)$ and $PM_{2}\left(G\right)$, respectively. We can see that, in the given domain, $PM_{1}\left(G\right)$ is more dominating than $PM_{2}\left(G\right)$.

**Figure 6:**
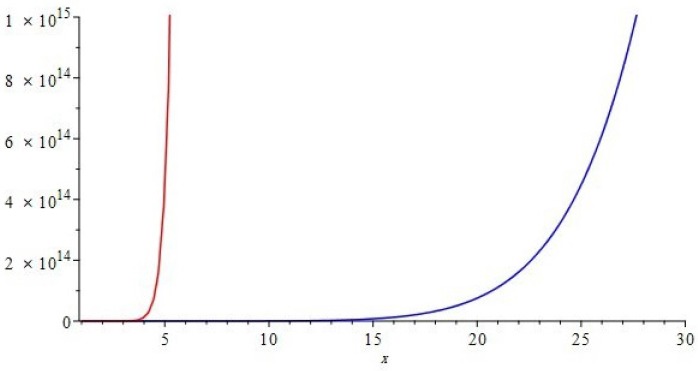
Comparison of first and second Zagreb polynomials $M_{1}\left(G,x\right)$ and $M_{2}\left(G,x\right)$ of G≅CG(m,n), for t=10=m=n. Blue and red represent $M_{1}\left(G,x\right)$ and $M_{2}\left(G,x\right)$, respectively. We can see that $M_{2}\left(G,x\right)$ grows more rapidly than $M_{1}\left(G,x\right)$.

**Figure 7:**
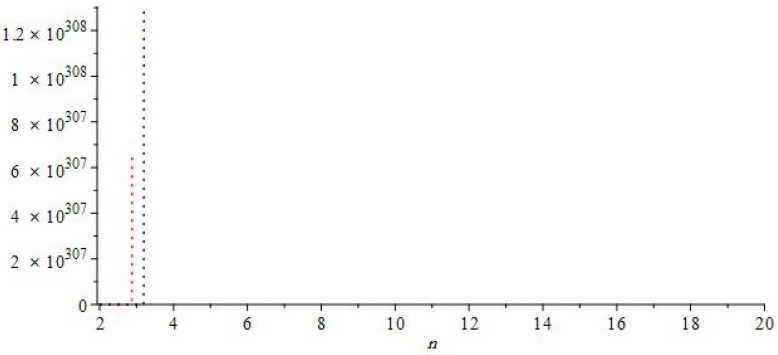
First and second multiple Zagreb indices $PM_{1}\left(G\right)$ and $PM_{2}\left(G\right)$ of *G* equivalent to CCC(n), for t=1. Blue and red colors represent $PM_{1}\left(G\right)$ and $PM_{2}\left(G\right)$, respectively. We can see that, in the given domain, $PM_{2}\left(G\right)$ is more dominating than $PM_{1}\left(G\right)$.

**Figure 8:**
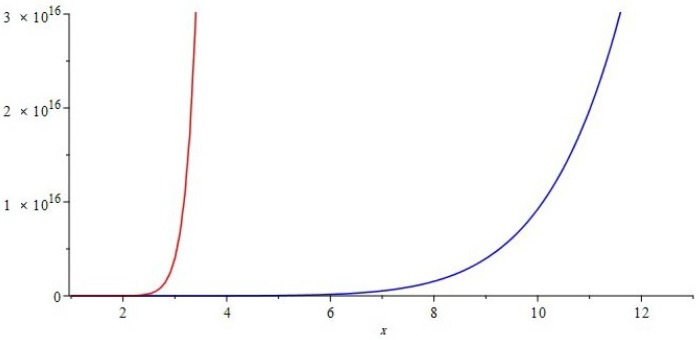
Comparison of first and second Zagreb polynomials $M_{1}\left(G,x\right)$ and $M_{2}\left(G,x\right)$ of G≅CCC(n), for t=10=m=n. Blue and red represent $M_{1}\left(G,x\right)$ and $M_{2}\left(G,x\right)$, respectively. We can see that $M_{2}\left(G,x\right)$ grows more rapidly than $M_{1}\left(G,x\right)$.

The graphical representations of first and second multiple Zagreb indices and Zagreb polynomials of CG(m,n) and CCC(n) are depicted in Figure 5, Figure 6 for certain values of m,n and Figure 7, Figure 8 for certain values of *n*, respectively. By varying the value of m,n in the given domain, the first, second multiple Zagreb indices and Zagreb polynomials behave differently. The comparison of first multiple Zagreb index, second multiple Zagreb index, and first and second multiplicative Zagreb indices $PM_{1}\left(G\right)$, $PM_{2}\left(G\right)$, $M_{1}\left(G,x\right)$ and $M_{1}\left(G,x\right)$ are depicted in Figure 5, Figure 6, Figure 7 and Figure 8.

## Figures and Tables

**Table 1:** Edge partition of CG(m,n) based on degree sum of end vertices of each edge.

(*d*(*s*), *d*(*t*))	Frequency
(2,2)	4
(2,3)	4(n+t-1)
(2,4)	4(nt+m-n-t)
(3,3)	4m+4t-10
(3,4)	6mn+6mt-14m-4n-6t+12
(4,4)	(4mn-3m-2n+1)t-7mn+5m+4n-2

**Table 2:** Edge partition of CCC(n) based on degrees of end vertices of each edge.

(*d*(*s*), *d*(*t*))	Frequency
(3,3)	$72\left(2^{3}-1\right)n^{-2}$
(3,4)	$24\left(2^{3}-1\right)n^{-2}$
(4,4)	$12\left(1+\sum_{r=3}^{n}2^{3}{\left(2^{3}-1\right)}^{r-3}\right)+8\sum_{r=0}^{n-2}{\left(2^{3}-1\right)}^{r}$
